# A New Approach to Enhancing Engagement in eHealth Apps

**DOI:** 10.2196/38886

**Published:** 2022-11-09

**Authors:** Ingrid Oakley-Girvan, John P Docherty

**Affiliations:** 1 Research, Value and Strategy Medable Inc Palo Alto, CA United States; 2 Weill Cornell Medical College White Plains, NY United States

**Keywords:** user engagement, eHealth, attrition, adherence, apps, app design, user experience

## Abstract

This viewpoint presents a 3-phase conceptual model of the process of user engagement with eHealth apps. We also describe how knowledge gleaned from psychosocial, behavioral, and cognitive science can be incorporated into this model to enhance user engagement with an eHealth app in each phase of the engagement process.

## Introduction

Effective user engagement is essential for the success of eHealth apps [1]. Yet, effective engagement with these apps remains a persistent problem [2]. Engagement tends to be highly variable and inconsistent [2-6], leading to problems in retention, data quality, and clinical impact [7,8]. Two factors may contribute to suboptimal user engagement with an eHealth app. The first is that a health care app differs from an app such as TikTok or Instagram. With TikTok and Instagram, the engagement systems consist primarily of providing people with more of what they already want [9]. In health care, people are asked to do things that they do not necessarily want to do. Someone may be very committed to losing weight but still want to eat cake. A person may be committed to participating in a clinical trial for many good reasons, but they may still not want to fill out a survey when tired or distracted by competing interests. To date, solutions to improve engagement have been offered but have had limited success [10-12].

A second factor is that sustained user engagement is a complex process [10]. Historically, engagement was defined by a variety of operational metrics; for example, the number of logins, number of pages visited, and number of tasks or modules completed [11]. However, these metrics do not capture or reflect the actual experience of the user [10,12-14]. More recently, the concept of engagement has been further differentiated from interface design and user experience. These two fields of knowledge contribute to the usability, ease, and pleasure of interacting with a digital technology and are important contributors to user engagement [15].

User engagement is characterized by attention, commitment, and involvement [12,14]. O’Brien and Toms [16] define engagement as “a quality of user experiences with technology that is characterized by challenge, aesthetic and sensory appeal, feedback, novelty, interactivity, perceived control and time, awareness, motivation, interest, and affect.” The resulting conceptual model of engagement distinguishes different phases of the engagement process: “Upon a point of engagement, the user initiates and sustains engagement in a task, he disengages, and potentially reengages several times with the system” [16]. These phases offer targets for interventions and content to enhance engagement and provide a useful structure for organizing and sequencing engagement-enhancing design.

## A Conceptual Model of Engagement

A conceptual model guiding the design and selection of interventions integrated into an eHealth app is needed to optimize patient engagement. A report by the Standing Committee for Psychology and Health and the E-Health Taskforce of the European Federation of Psychologists’ Associations stated, “Utilization of a theoretical design framework in digital intervention planning cultivates and maintains user engagement and motivation to adhere to the intervention throughout its intended duration. Examining the literature on digital interventions suggests that most digital programs evaluated are not rooted in specific theoretical frameworks” [12]. A coherent, theory-based model draws upon established bodies of psychosocial, behavioral, and cognitive science to enhance the process, depth, and consistency of patient engagement with eHealth apps. This viewpoint describes and illustrates a model structured around 3 phases of the engagement process: initiation, strengthening, and maintenance incorporating knowledge from psychosocial, behavioral, and cognitive science.

### Initiation of Engagement

Three components of initiation can be informed by the aforementioned science: (1) design of the app’s user experience; (2) decision and intent to participate; and (3) technical competence, digital anxiety, and health literacy.

#### Design

An effective user interface design is essential for both inducing participation and reducing barriers and friction points that can impede participation. Through better design, the user experience is enhanced and engagement is increased. The 5 Principles of Intentional Design [17], Rogers’ 5 attributes of product perception, and Rogers’ 5-category model of adopter types and innovation diffusion [18] are established bodies of psychosocial knowledge that provide practical guidance for effective app design.

#### Decision and Intent to Participate

This is informed by 3 relevant bodies of work—Prochaska and DiClemente’s [19] transtheoretical model of Stages of Change, Motivational Interviewing [20], and Hibbard et al’s [21] Patient Activation Measure.

#### Technical Competence, Digital Anxiety, and Health Literacy

Barbeite and Weiss’ [22] model of digital anxiety and technical self-efficacy directly informs the initiation of engagement. This model posits that the ability to competently use a digital app has 2 aspects: (1) an actual technical competence to operate the digital app and its associated device and (2) a subjective sense of anxiety, usually driven by the fear of making an app-disabling mistake. From an information-processing perspective, the negative feelings associated with high anxiety detract cognitive resources from task performance. Similarly, health literacy—the ability to understand the information that the app provides and respond accurately and completely where required—should be assessed and improved as needed. A large body of research supports the importance of adequate health literacy for effective user engagement [23].

### Strengthening of Engagement

After engagement has been initiated, 2 well-studied processes can strengthen engagement: (1) the therapeutic alliance [24] and (2) behavioral conditioning to convert controlled processes that require conscious thought to automatic processes [25].

#### A Digital Analog of the Therapeutic Alliance

The therapeutic alliance in psychotherapy is characterized by a relationship that is collaborative in nature and characterized by a positive affective bond between the patient and the therapist [26]. It is further characterized by a relationship in which the therapist and patient agree on the treatment’s goals and tasks [27]. Establishing a positive therapeutic alliance is essential for successful psychotherapy, even apart from the type of psychotherapy or specific technical competence of the therapist [28]. Fortunately, much is known about how to establish and deepen such a therapeutic alliance [29-32]. Attention to these alliance-forming and deepening factors in the design and content of the eHealth experience transforms the affective nature of the patient’s experience, engages the patient as a collaborator, and establishes clear agreement on the mutual and respective roles and tasks of the patient and the eHealth system. Such a relationship is a powerful motivator for the patient to remain engaged.

#### Behavioral Conditioning and Automaticity

The basic principles of behavioral conditioning, including both primary and secondary reinforcement to promote positive engagement behaviors and to transform consciously directed study-specific tasks into automatic habits, can be useful to deepen engagement. This transformation relieves the patient of cognitive burden and eases completion of study tasks. A converging body of work offers complementary methods to achieve this transformation, including habit theory [33-35], dual-process theory, and an understanding of the neurobiology of this transformation [25].

### Maintenance of Engagement

Some factors can interfere with a patient’s continued engagement with the eHealth app and result in missing tasks, sporadic participation, or complete attrition. These include boredom, fatigue, other demands of life, and other intercurrent events [36]. Psychological and social science provides possible remedies to help maintain engagement.

#### Stress Management

It has been demonstrated that intercurrent stress can interfere with a previously successful level of patient engagement [37]. Basic activities for stress self-management are well established and can be accessed as needed through the study app [38].

#### Adherence Management

The literature on health care adherence offers common reasons for nonadherence and describes interventions built to support continued adherence [39]. This work is relevant to ways to prevent attrition and retain engagement. For example, one successful intervention is a continuous adherence-monitoring system that identifies lapses in task completion and notifies the patient and treatment team of such lapses and of the opportunity for situation-specific intervention [40,41]. Such a system engages the patient at a point of possible disjuncture and can be readily implemented in an eHealth app.

#### Nudge Theory

Nudge theory [42] provides an understanding of human choice derived from behavioral economics and a demonstration of the effect of the choice environment on the decisions an individual makes. In the technology sphere, this is referred to as the choice architecture of the app design and its associated functions to help guide a user to a beneficial choice. Embodiments of nudge theory include recommender systems, reminder systems, and motivational messaging [43].

## Assessing the Model

This viewpoint proposes that each implementation of these knowledge-based strategies be systematically tested to assess its individual contribution to improving user engagement before being introduced into the final eHealth app design using a standardized assessment such as O’Brien et al’s [44] User Engagement Scale Short Form questionnaire. For example, in our own desk research work, we have developed a preliminary module to assess health literacy, and we have developed a library of educational materials that can be provided as needed to help the user achieve adequate literacy to successfully use a particular eHealth app. We propose to test this module and each subsequent module following the strategy described above before incorporating it into the final app design. We then propose to test an app with these enhancements against an app without them.

## Conclusion

This conceptual model draws upon an extensive body of literature on behavioral, cognitive, and psychosocial science with the aim of improving the extent, quality, and clinical impact of user engagement with eHealth apps at each of the 3 major phases of the engagement process.
